# Complete Genome Sequences of Four Canadian Mycoplasma bovis Strains Isolated from Bison and Cattle

**DOI:** 10.1128/MRA.00136-21

**Published:** 2021-04-29

**Authors:** Harish Menghwar, Tracy Prysliak, Jose Perez-Casal

**Affiliations:** a Vaccine and Infectious Disease Organization (VIDO), University of Saskatchewan, Saskatoon, Canada; University of Arizona

## Abstract

Mycoplasma bovis is a major bacterial pathogen that causes respiratory diseases in cattle and bison. We report here the complete genome sequences of four Mycoplasma bovis strains isolated in three Canadian provinces. These genome sequences could provide important information on virulence factors and targets for new vaccines against M. bovis.

## ANNOUNCEMENT

Mycoplasma bovis was first identified from mastitic milk in the United States in 1961 (1). M. bovis is the most prevalent bovine mycoplasma pathogen, causing pneumonia, otitis media, arthritis, mastitis, and reproductive disorders, resulting in unprecedented economic problems in both the dairy and beef industries globally (2, 3). M. bovis has also emerged as an important pathogen of North American bison (Bison bison) (4). The M. bovis factors responsible for disease development are not known; thus, genomic sequencing of different isolates of this pathogen may help in the elucidation of these factors. Our previous study showed differences in the modulation of peripheral blood mononuclear cell (PBMC) proliferation, invasion of tracheal and lung epithelial cells, along with modulation of apoptosis and survival in alveolar macrophages between some M. bovis cattle and bison isolates (5). We performed genomic sequencing on four strains, two isolated from cattle and two from bison, as a first step toward identifying the differences between these genomes that may explain their diverse phenotypes.

In this study, we obtained the complete genome sequences of M. bovis strains Mb1, Mb160, Mb300, and Mb304, isolated from clinical cases in bison and cattle in Saskatchewan, Alberta, and Manitoba, Canada (5). The details of M. bovis isolates and the genome characteristics are provided in Table 1. The isolates were grown from frozen stocks in modified Hayflick’s broth (HFB; BD Difco, France), supplemented with 20% heat-inactivated horse serum (Gibco, Life Technologies, USA), 0.1% yeast extract (BD Bacto, France), and 0.1 mg/ml ampicillin (Bio Basic Canada, Inc.), and incubated at 37°C in 5% CO_2_ for 48 h. The strains were single-cell cloned by passaging through a 0.2-μm syringe filter, serially diluted, plated onto Hayflick’s agar (HFA) plates, incubated at 37°C in 5% CO_2_ for 5 days, and identified by PCR targeting the *uvrC* gene of M. bovis (6). The purified isolates were cultured in Hayflick’s broth containing 50% glycerol and stored at −80°C.

**TABLE 1:** Name, geographic location, anatomic site, date of origin, sequencing metrics, and accession numbers of M. bovis isolate genome sequences

Isolate name	Geographical origin (province)	Anatomic site of isolation	Yr of origin	Host species	Genome length (bp)	No. of reads from:		Genome coverage (×)	PacBio read *N*_50_ (bp)	G+C content (%)	Total no. of CDS^*a*^	No. of pseudogenes	No. of RNA genes	SRA accession no. for:		GenBank accession no.
						PacBio	Illumina							PacBio reads	Illumina reads	
Mb1	Saskatchewan	Stifle joint	2006	Cattle	978,001	321,573	4,237,973	872.32	16,921	29.30	812	42	43	SRX9572988	SRX9572984	CP069056
Mb160	Alberta	Lung	2008	Cattle	1,049,056	190,447	3,748,153	410.05	18,114	29.26	854	64	43	SRX9572989	SRX9572985	CP069057
Mb300	Saskatchewan	Lung	2012	Bison	1,162,329	298,524	4,824,606	990.91	16,996	29.01	947	82	43	SRX9572990	SRX9572986	CP069100
Mb304	Manitoba	Lung	2012	Bison	1,039,622	253,858	4,465,692	961.16	18,644	29.64	843	56	43	SRX9572991	SRX9572987	CP069101

a CDS, coding DNA sequences.

Genomic DNA was extracted for both PacBio and Illumina sequencing by using the DNeasy UltraClean microbial kit (Qiagen). The harvested DNA was detected by agarose gel electrophoresis and quantified using a Qubit 2.0 fluorometer (Thermo Scientific). Genomic sequencing was carried out by CD Genomics (New York, USA). The libraries were constructed using the SMRTbell DNA template with a fragment size of >10 kb, selected using a BluePippin system (7). The library quality was analyzed by Qubit and quantitative PCR (qPCR), and the average fragment size was estimated using an Agilent 2100 Bioanalyzer. The whole genome was sequenced using the PacBio Sequel II platform. Default parameters were used for all software. Microbial assembly of the sequencing reads was performed using HGAP4 and Canu v1.6 software (8). The Canu v1.6 assembly software for PacBio was used to correct the reads, trim the ambiguous regions, and finally assemble high-quality genome sequences. Multiple assembled results were mirrored to each other to generate completed genome sequences.

Illumina sequencing was carried out using the NovaSeq 6000 platform with the 2 × 150-bp sequencing configuration (CD Genomics). For the next-generation sequencing (NGS) data or Illumina reads, Pilon v.1.22 (9) was used for assembly and correction based on the NGS reads to obtain the final assembly result. The sequences reported here provide valuable information for future studies on M. bovis virulence factors and targets for vaccines. The genomes were annotated by NCBI using the Prokaryotic Genome Annotation Pipeline (PGAP) v4.2 (https://www.ncbi.nlm.nih.gov/genome/annotation_prok/) (10).

### Data availability.

These genome assemblies have been deposited in GenBank under BioProject accession number PRJNA680748, with the isolate-specific accession numbers indicated in Table 1. The raw sequence reads have been deposited in the Sequence Read Archive (SRA) under the accession numbers listed in Table 1.
